# Metallic nanoparticle-coated sutures: a breakthrough in the field of surgery

**DOI:** 10.1097/MS9.0000000000000162

**Published:** 2023-02-17

**Authors:** Abdul Moiz Khan, Syeda Shahnoor, Aliza Sajjad

**Affiliations:** a Ayub Medical College, Abbottabad; b Dow University of Health Sciences, Karachi; c Peshawar Dental College, Peshawar, Pakistan

*Dear Editor,*

Surgical site infections (SSIs) are postoperative infection manifestations at the site of surgery performed. SSI is prevalent at a high rate of 1.2–5.2% in developed countries^1^. Amongst hospital-acquired infections, SSIs are the most costly and common, with a 20% presentation^2^. SSIs increase mortality risk by 2–11 folds and extend hospital stay by 9.7 days^2^. Patients with SSIs are vulnerable to a higher risk of hospital readmissions, longer ICU stays, and various other postoperative complications, thus not only causing financial stress on the patients but also burdening the economy and healthcare system of the country^1^. SSI occurs due to microbial invasion and pathogenesis at the surgical site. It mostly begins with contamination by local microflora or environmental pathogens. *Staphylococcus aureus*, *Escherichia coli*, *Pseudomonas aeruginosa*, and *Citrobacter* species are the common bacteria involved in SSIs^3^. Duration of surgery, length of preoperative hospital stays, preexisting infections, and diabetes are some of the leading risk factors for nurturing microorganisms that lead to SSIs^1^. The presence of any sort of foreign materials, like surgical implants and dressing on a wound, provides an anchoring surface for bacterial invasion^1^. Similarly, sutures also act as prosthetic implants and biofilms have been identified to form over them. They provide conductive material for the adherence and colonization of infection-causing agents. Therefore, sutures coated with a wide-range antibiotic like triclosan started being used for surgeries, preventing it from becoming a nidus for biofilm and infection^4^. However, unfortunately, these antibiotics are progressively losing their effectiveness due to antibiotic resistance.

In recent years, studies around metallic nanoparticles (NPs) have been the topic of interest because NPs not only exhibit antibacterial properties but are also nontoxic to mammalian cells, and bacteria develop less resistance to metals^5^. The possibilities of applying metallic NPs to sutures have also been explored. In the latest work by Vieira *et al*.^6^, a novel technique was developed to successfully coat different metallic NPs on surgical sutures, and their antibacterial potentials were studied. Synthetic absorbable PDS-II (polydioxanone) sutures made of polyester were used for this study. Metallic NPs were then coated on these sutures by the dip-coating method. Different aqueous solutions were prepared for each metal NP (ZnO, TiO_2_, Fe_2_O_3_, Cu, Cu_2_O, and MgO) by taking 25 ml of ultrapure water and adding 0.1 M Na_2_SO_4_, 30 mM of ascorbic acid and the desired NP in it. The PDS-II sutures were then individually dipped in liquid while the solution was continually agitated mechanically. A thin layer of silk fibroin was then applied to prevent the detachment of NPs. This method of the coating was analyzed to preserve the strength, elasticity, and degradability of PDS-II sutures. The success of the experiment was based on calculations analyzing the antibacterial properties against *P. aeruginosa* and *S. aureus*, and the extent of minimal cytotoxicity that was exhibited by metallic NP-coated sutures. Metallic NP coatings displayed strong antibacterial properties, as indicated by the percentage of bacteria killed, which varied from 40% for MgO-coated sutures to about 90% for Fe_2_O_3_, compared to 15% for bacteria killed by uncoated PDS-II sutures after 7 days. Also positively, all sutures displayed minimal cytotoxicity, that is cell viability greater than 70%, supporting the hope of metallic NP-coated sutures being used as potentially viable postoperative antibacterial therapy.

Innovations are always of prime importance in the field of medicine. Over the years, developments have been made to prevent SSIs. Metallic NP-coated sutures are highly effective against SSIs, cost-friendly, and easily applicable. Because of the promising results displayed, further advanced research should be conducted on its viability so that the complications caused by SSIs can be minimized. If additional research supports the efficiency of metallic NP-coated sutures, they would be a promising breakthrough.

## Ethical approval

Not required.

## Patient consent

Not needed.

## Sources of funding

Not applicable.

## Author contribution

All authors equally contributed.

## Conflicts of interest disclosure

There are no conflicts of interest.

## Research registration unique identifying number (UIN)

None.

## Guarantor

Syeda Shahnoor, Department of Internal Medicine, Dow University of Health Science, Karachi, Pakistan. E-mail: syedashahnoor17@gmail.com
